# GelMA Hydrogel Stiffness Modulates IL-6- and BMP-2-Induced Immune Dysregulation in Human Mesenchymal Stem Cells

**DOI:** 10.3390/biomedicines14061193

**Published:** 2026-05-25

**Authors:** Tony D. Baldini, Soren D. Johnson, Aneesh S. Bhat, Mengyao Liu, Andrea C. Filler, Mark A. Lee, J. Kent Leach, Maryam Rahmati, Augustine M. Saiz

**Affiliations:** 1 Department of Orthopaedic Surgery, UC Davis Health, 4860 Y Street, Suite 3800, Sacramento, CA 95817, USA; 2 College of Medicine, California Northstate University, 9700 W Taron Drive, Elk Grove, CA 95757, USA; 3 Department of Biomedical Engineering, UC Davis, Davis, CA 95616, USA

**Keywords:** interleukin 6, inflammation, bone morphogenetic protein, gelatin methacryloyl (GelMA), fracture healing

## Abstract

**Background**: Fracture healing requires a coordinated inflammatory response, and its dysregulation, as seen in polytrauma, can impair bone regeneration. Human mesenchymal stem cells (hMSCs) play a central role in fracture repair through osteogenic differentiation and also via their secretome, which regulates local inflammation, angiogenesis, and tissue regeneration. Interleukin-6 (IL-6), an early pro-inflammatory cytokine, contributes to fracture healing by promoting MSC recruitment and osteogenic differentiation, whereas bone morphogenetic protein-2 (BMP-2) is a key osteoinductive factor that drives bone formation. However, the combined effects of IL-6 and BMP-2 on the hMSC secretome remain poorly understood. **Methods**: We cultured hMSCs in osteogenic media supplemented with recombinant IL-6 (1–20 ng/mL) alone or combined with recombinant BMP-2 (1 ng/mL) on tissue culture plastic (TCP) and within gelatin methacryloyl (GelMA) hydrogels of low (~3 kPa), medium (~15 kPa), and high (~30 kPa) stiffness. Osteogenic differentiation was assessed by alkaline phosphatase (ALP) activity and calcium deposition; cytokine profiling was performed using a multiplex antibody array. **Results**: When cultured on TCP, IL-6 suppressed ALP activity by day 21. Co-treatment with IL-6 and BMP-2 induced a dysregulated secretome with concurrent upregulation of pro-inflammatory markers (MIP-1α, TNF-α, and GM-CSF) and anti-inflammatory mediators (IL-10, TGF-β1, and VEGF). This hyperinflammatory response was attenuated when hMSCs were encapsulated in GelMA, with high-stiffness gels most effectively suppressing pro-inflammatory chemokines and medium-stiffness gels yielding the highest ALP activity. **Conclusions**: These findings suggest that mechanically tuned GelMA hydrogels modulate immune and osteogenic responses of hMSCs in vitro, warranting further investigation in the context of scaffold design for fracture care.

## 1. Introduction

Fracture healing is a highly regulated process involving sequential stages of inflammation, repair, and remodeling [1]. The immediate activation of the inflammatory phase, which involves the influx of cytokines and leukocytes to the fracture site following initial hematoma formation, is crucial to the eventual restoration of mechanical and structural integrity to the affected bone [2]. Equally crucial is the shift towards a largely anti-inflammatory local environment in the repair and remodeling stages, as neutrophils undergo apoptosis by macrophages and anti-inflammatory cytokines induce the polarization of macrophages from the pro-inflammatory M1-like phenotype to the anti-inflammatory M2-like phenotype [2,3]. Dysregulation of these immune responses, such as that observed in the sustained hyperinflammation of polytrauma patients, significantly disrupts this process [4]. For instance, the incidence of heterotopic ossification, fracture non-union, or delayed healing is increased when the infiltration of pro-inflammatory cytokines and leukocytes is prolonged [5,6,7]. Therefore, it is vital to expand our understanding of how inflammation is controlled to manage and promote fracture healing in the clinic.

Interleukin-6 (IL-6), a key signaling molecule implicated in the regulation of bone healing, is a pleiotropic cytokine that peaks in concentration around six hours post-fracture. IL-6 plays a key role in immune activation through formation of the IL-6/IL-6 receptor complex, allowing binding to the transmembrane protein gp130 and promoting gene transcription primarily through the STAT3 signaling pathway [8,9]. IL-6 also plays a complex role in the regulation of osteogenesis, as its expression has been implicated in both promoting the osteogenic differentiation of bone marrow-derived mesenchymal stem cells (BM-MSCs) and inhibiting osteoblastic gene expression in MC3T3-E1 cells [10,11]. This dual role is likewise observed in the inflammatory function of IL-6. IL-6 signaling operates through both the anti-inflammatory classic signaling pathway, in which the IL-6 receptor is membrane-bound (mIL-6R), and the pro-inflammatory trans signaling pathway, in which the receptor is soluble (sIL-6R) [12]. The balance of these contrasting roles is largely dependent on the interaction of IL-6 with other signaling proteins, such as bone morphogenetic proteins (BMPs).

BMP-2 is a potent osteoinductive growth factor that is used therapeutically for the promotion of bony tissue regeneration. However, its increased clinical use has revealed significant inflammatory side effects, largely through increasing the concentration of pro-inflammatory cytokines [13]. Additionally, the interaction between BMP-2 and IL-6 worsens these inflammatory side effects, further detracting from the osteogenic potential of BMP-2 therapies [13]. Therefore, clinical strategies addressing the management of this inflammation while maintaining bone healing are of the utmost importance.

Gelatin methacryloyl (GelMA)-based hydrogels are widely used biomaterials for the study of mechanoregulation, where the mechanical stimulation of damaged tissue is used to promote tissue regeneration by leveraging the mechanosensitive nature of mesenchymal stem cells (MSCs) [14,15]. GelMA hydrogels can be tuned across a wide range of stiffnesses (~3–40 kPa) that significantly influence cell behavior, including stem cell differentiation, fibroblast activation, and tissue-specific regeneration outcomes [16,17,18]. Softer substrates (~0.1–1 kPa) favor neurogenic differentiation, intermediate stiffness (~8–17 kPa) promotes myogenic differentiation, and stiffer matrices (~25–40 kPa) support osteogenic differentiation [19,20,21,22]. Altering matrix stiffness has also been shown to influence cytokine secretion. Stiffer matrices promote the secretion of pro-inflammatory cytokines like TNF- α and drive the polarization of macrophages toward the M1-like phenotype, further increasing pro-inflammatory cytokine secretion [3], while the secretion of inflammatory cytokines is reduced in MSCs on softer substrates [23]. Therefore, fine tuning matrix stiffness may attenuate inflammatory hyperactivation. The GelMA stiffnesses evaluated in this study (3, 15, and 30 kPa) capture the favorable osteogenic differentiation range while exploring softer matrices to potentially elicit favorable secretory effects.

Although IL-6 and BMP-2 have been well-studied individually in settings of inflammation and osteogenesis, elucidating their combined effect on the hMSC secretome would provide crucial insight into the molecular basis of inflammatory dysregulation in polytrauma. Furthermore, it is unknown whether variations in the stiffness of GelMA hydrogels can attenuate the inflammatory response of these proteins. To address this gap, this study investigated the relationship between GelMA hydrogel stiffness and the effects of IL-6 and BMP-2 on MSC cytokine profile using an in vitro cell culture model. We hypothesized that hydrogel stiffness would influence the IL-6/BMP-2-induced hyperinflammation observed in hMSCs, promoting the osteogenic differentiation observed in proper fracture healing. By characterizing the osteogenic and inflammatory responses to IL-6 and BMP-2 and determining the degree to which variations in gel matrix stiffness affect immunomodulation, this study aims to guide development of biomaterial scaffolds used in the management of fracture healing in polytrauma patients.

## 2. Materials and Methods

### 2.1. In Vitro Assays

#### GelMA Hydrogel Synthesis

Lyophilized GelMA (300 g bloom; 80% degree of substitution (DS); MilliporeSigma, Burlington, MA, USA) was dissolved at 10% (*w*/*v*) in alpha-minimum essential medium (α-MEM) containing 0.15% or 0.30% (*w*/*v*) Irgacure 2959 (IG, MilliporeSigma) at 60 °C. Irgacure solutions were filtered through a 0.22 μm polyethersulfone membrane prior to mixing with GelMA. GelMA solutions were pipetted into 4 mm circular silicone molds and exposed to UV light (320–500 nm, OmniCure S2000, Excelitas Technologies, Mississauga, ON, Canada) at 10 mW/cm^2^. Gel groups with different stiffnesses were fabricated by varying the Irgacure concentration and UV exposure time: 0.15% IG—1 min (~3 kPa, low), 0.15% IG—2 min (~15 kPa, medium), and 0.30%—5 min (~30 kPa, high).

### 2.2. Compressive Moduli

A cylindrical acellular hydrogel was fabricated and equilibrated in media overnight at 37 °C prior to mechanical testing. A single-column testing system (Instron 3345, Norwood, MA, USA) was used to compress acellular GelMA gels at 0.05 mm/s (strain rate of ~3.33%/s). The diameter of each gel was measured prior to testing to normalize the force for each sample. Compressive moduli were determined from the slope of a linear regression fit between 5 and 10% strain on a stress–strain curve. A minimum of three gels per group were tested (*n* ≥ 3).

### 2.3. Cell Culture

Human mesenchymal stem cells (hMSCs; Rooster Bio, Frederick, MD, USA) were expanded on tissue culture plastic (TCP) in growth media (GM) consisting of α-MEM supplemented with 10% fetal bovine serum (FBS; GenClone, Genesee Scientific, San Diego, CA, USA) and 1% penicillin–streptomycin (Gemini Bio Products, West Sacramento, CA, USA) under standard conditions (37 °C, 5% CO_2_, and a humidified atmosphere). Osteogenic differentiation media (OMs) were prepared by supplementing GM with 0.01 μM dexamethasone, 50 μg/mL L-ascorbic acid 2-phosphate, and 10 mM sodium β-glycerophosphate. hMSCs were passaged serially until passage 5 (P5), with media changes every 48 h and passaging at approximately 80% confluence.

For monolayer experiments, P5 hMSCs were seeded at 2.0 × 10^4^ cells per well in 24-well plates in 1 mL of OM. The cells were treated with recombinant human IL-6 (PeproTech, Cranbury, NJ, USA) at 1, 10, or 20 ng/mL or with a vehicle control (OM alone). To evaluate the effect of BMP-2 on IL-6-induced responses, a subset of wells treated with IL-6 at 1 ng/mL was additionally supplemented with recombinant human BMP-2 (R&D Systems, Minneapolis, MN, USA) at 1 ng/mL. OM was refreshed every 48 h throughout all experiments. Cultures were maintained for 3, 10, or 21 days, depending on the endpoint assessed.

For hydrogel experiments, P5 hMSCs were encapsulated in GelMA at a 1.25 × 10^6^ cells/mL and crosslinked as described above. Encapsulated cells were cultured in OM with IL-6 (1 ng/mL) or IL-6 (1 ng/mL) + BMP-2 (1 ng/mL) alongside gel-only OM controls for 21 days with media refreshed every 48 h.

### 2.4. Viability Assay and DNA Concentration

Cell viability was assessed using live/dead staining. Briefly, live cells were labeled with calcein-AM (2 μM; green fluorescence, intracellular esterase activity; Thermo Fisher Scientific, Waltham, MA, USA) and dead cells with propidium iodide (5 μM; red fluorescence, loss of membrane integrity; Thermo Fisher). Fluorescent images were acquired using a confocal laser scanning microscope (Leica TCS SP8, Leica Microsystems, Wetzlar, Germany). Total DNA content was quantified using the Quant-iT PicoGreen dsDNA Assay Kit (Thermo Fisher) per the manufacturer’s instructions. Fluorescence was measured at excitation/emission of 485/528 nm using a Synergy HTX plate reader (BioTek Instruments, Winooski, VT, USA). DNA concentrations were calculated from the standard curve with correction for sample dilution.

### 2.5. Osteogenic Differentiation

Intracellular alkaline phosphatase (ALP) activity was measured at days 3, 10, and 21 for IL-6 monolayer groups, and at day 21 for IL-6 + BMP-2 and GelMA groups, using a colorimetric assay based on the hydrolysis of p-nitrophenyl phosphate (pNPP; Sigma-Aldrich). Cell lysates were prepared as described above. ALP activity was normalized to the total DNA content per sample.

On day 21, mineralization was assessed by quantifying calcium deposition. Cell layers and hydrogels were completely dissolved in 1 M hydrochloric acid (HCl) at 60 °C for 48 h. Calcium content was measured using the Calcium Liquid Reagent Diagnostic Kit (Stanbio Laboratory, Boerne, TX, USA) per the manufacturer’s protocol and normalized to total DNA content.

### 2.6. Cytokine Profiling

To characterize the inflammatory secretome, conditioned media were collected from hMSC cultures at day 21 to align with the terminal osteogenic differentiation endpoint, as this time point is standard for assessing mature osteogenic outcomes (mineralization and sustained ALP activity). Day 21, therefore, allowed direct correlation between the MSC secretome and the concurrent osteogenic differentiation status of the cells. Following a 48 h preconditioning period in fresh OM, the media were changed and refreshed every 48 h for the duration of the experiment. At day 21, conditioned media were harvested and stored at −80 °C until analysis. Secreted cytokine and growth factor levels were quantified using a human cytokine antibody array (C-Series 1000; RayBiotech, Inc., Norcross, GA, USA), according to the manufacturer’s protocol. For each experimental group, media from *n* = 6 wells were pooled prior to array analysis. Array signals were normalized to positive control spots included on each membrane, and relative expression was visualized as a heat map.

### 2.7. Statistical Analysis

Statistical analyses were performed using GraphPad Prism version 9.5.1 (GraphPad Software, San Diego, CA, USA). Data normality was assessed using the Kolmogorov–Smirnov test. Normally distributed data are reported as means ± standard deviation; non-normally distributed data are reported as medians with interquartile ranges. For comparisons among three or more groups, one-way or two-way ANOVA was applied to normally distributed data, and the Kruskal–Wallis test on ranks was used when normality assumptions were not met. Post hoc pairwise comparisons were performed with appropriate corrections for multiple comparisons. Statistical significance was defined as *p* < 0.05.

## 3. Results

**IL-6 promotes early alkaline phosphatase expression but suppresses late osteogenic activity in human mesenchymal stem cells.**

**Figure 1:**
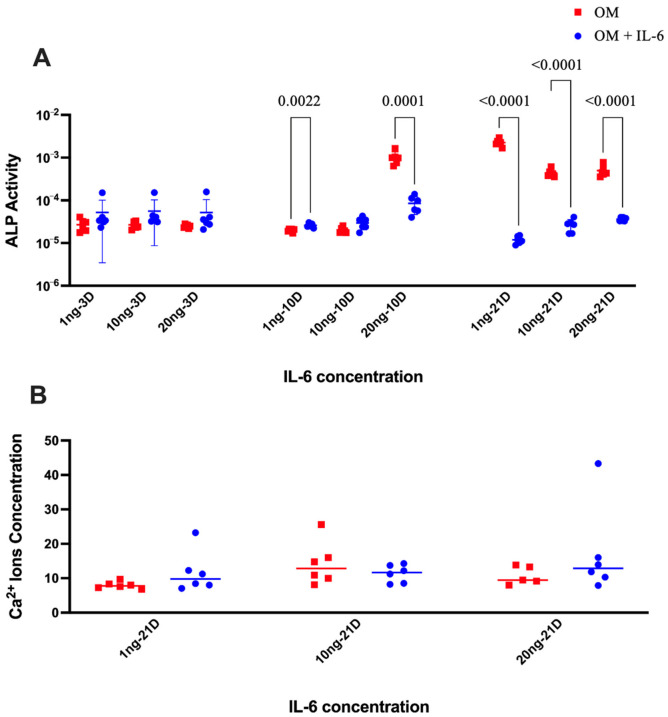
IL-6 enhances early alkaline phosphatase (ALP) expression in human mesenchymal stem cells (hMSCs) but suppresses it at later stages (day 21) without affecting calcium levels. (**A**) Relative expression of ALP from hMSCs treated with osteogenic media (OMs) and 1, 10, or 20 ng/mL of IL-6 after 3, 10, and 21 days. (**B**) Calcium levels after hMSC treatment with osteogenic media and 1, 10, or 20 ng/mL of IL-6 at 21 days. Data are means + SD (*n* = 6).

Figure 1A illustrates the alkaline phosphatase (ALP) expression in hMSCs after exposure to different IL-6 concentrations and time points, with ALP serving as a marker of early osteogenic differentiation of MSCs. At 10 days, hMSCs stimulated with 1 ng/mL of IL-6 exhibited increased ALP expression compared with osteogenic media, while hMSCs stimulated with 20 ng/mL of IL-6 had lower ALP activity. By day 21, ALP expression in hMSCs was suppressed by all concentrations of IL-6 compared with osteogenic media.

Figure 1B displays the calcium ion concentration following MSC treatment of IL-6 at 21 days after exposure. We did not observe any measurable changes in calcium ion concentration compared with osteogenic media controls for cells stimulated with IL-6 at any dosage. An IL-6 concentration of 1ng was used for the rest of this study, given that MSC exposure to all three IL-6 concentrations produced similar ALP and calcium levels at each respective time point.

**Co-treatment with IL-6 and BMP-2 increases expression of both pro- and anti-inflammatory markers in human mesenchymal stem cells (hMSCs).**

**Figure 2:**
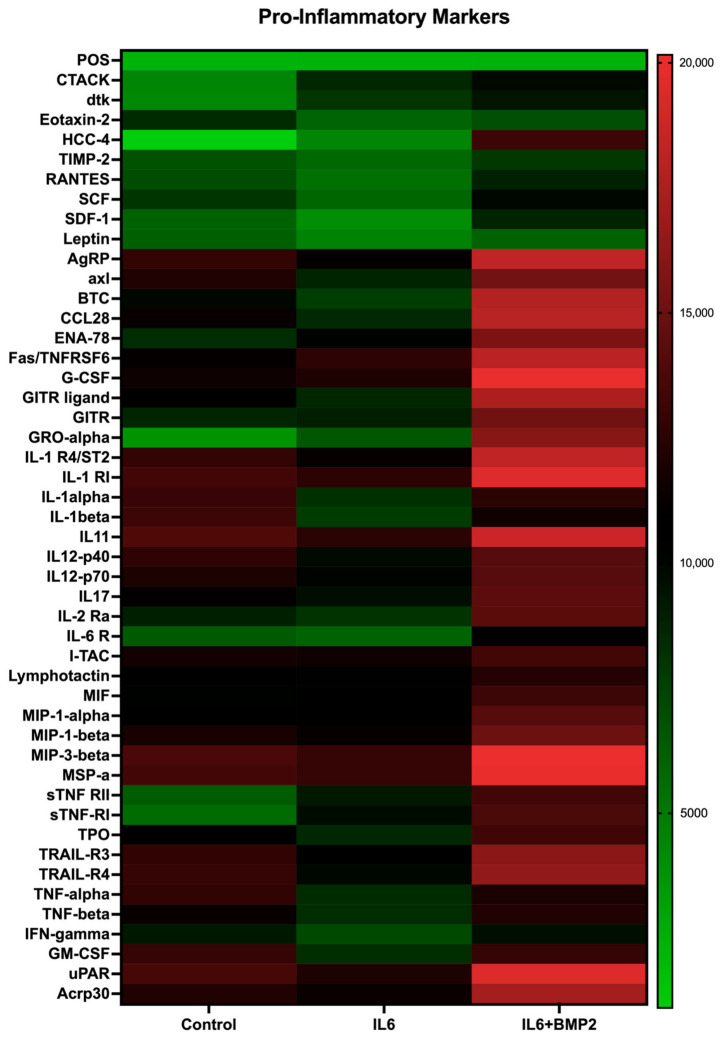
The combination of IL-6 and BMP-2 increases the pro-inflammatory effects on human mesenchymal stem cells (hMSCs) in vitro. A heat map of the secretome protein expression of inflammatory cytokines. *N* = 6; samples were pooled together for each group. Green color depicts low levels, and red depicts high levels of expression. POS = positive control spots, which were used as the internal control/baseline.

Figure 2 highlights the impact of IL-6 and BMP-2 on the inflammatory secretome profile of MSCs treated with osteogenic media after 21 days. We observed distinct changes in the secretion of pro-inflammatory cytokines across three experimental groups: MSCs exposed to osteogenic media alone, MSCs cultured with IL-6, and MSCs cultured with the combination of IL-6 and BMP-2. Key pro-inflammatory chemokines, including macrophage inflammatory protein-1 alpha (MIP-1-alpha), MIP-1-beta, and MIP-3-beta, were upregulated in the IL-6 + BMP-2 group. Moreover, addition of BMP-2 increased the expression of classic inflammatory cytokines IL-1 and TNF-alpha (*p* < 0.001) in comparison with IL-6 alone. Granulocyte macrophage colony-stimulating factor (GM-CSF), which stimulates production of neutrophils and monocytes, was also upregulated in the IL-6 + BMP-2 group, representing a skew towards innate immunity, favoring inflammation. These results suggest that combined IL-6 and BMP-2 treatment exerts additive pro-inflammatory effects on the MSC secretome in vitro.

**Figure 3:**
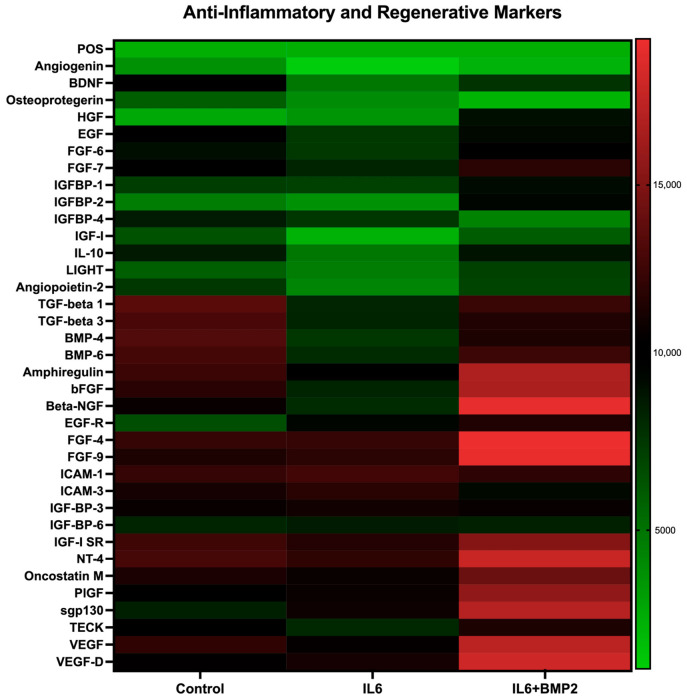
IL-6 suppresses anti-inflammatory markers on human mesenchymal stem cells (hMSCs) in vitro, and addition of BMP-2 increases expression of anti-inflammatory markers. A heat map of the secretome protein expression of inflammatory cytokines. *N* = 6; samples were pooled together for each group. Green color depicts low levels, and red depicts high levels of expression. POS = positive control spots, which were used as the internal control/baseline.

Figure 3 underscores the impact of IL-6 and BMP-2 on the anti-inflammatory and regenerative secretome profile of MSCs treated with osteogenic media at 21 days. While IL-6 exposure decreased MSCs’ expression of anti-inflammatory markers, addition of BMP-2 substantially elevated these markers in comparison with IL-6 alone. Notable immunosuppressive cytokines upregulated with IL-6 and BMP-2 included IL-10 (*p* < 0.001), transforming growth factor beta 1 (TGF-β1), and TGF-β3. Similarly, angiogenesis-related factors such as vascular endothelial growth factor (VEGF) were upregulated when MSCs were exposed to both IL-6 and BMP-2. Taken together, these data indicate that IL-6 and BMP-2 co-treatment enhances both pro- and anti-inflammatory signaling in MSCs compared with IL-6 treatment alone and controls, consistent with immune dysregulation.

**GelMA hydrogels support MSC viability, and DNA content and ALP activity are consistent between monolayer culture treatment groups.**

**Figure 4:**
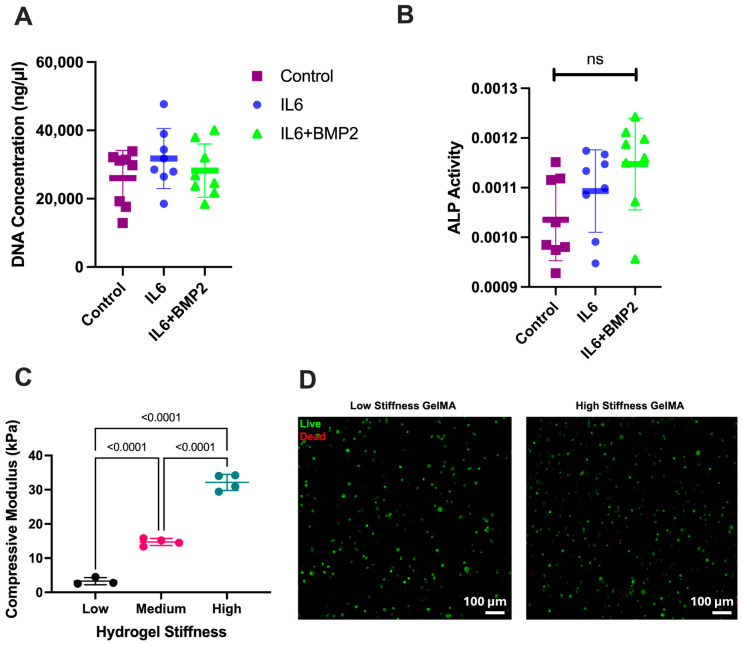
Both medium- and high-stiffness gelatin methacryloyl (GelMA) are biocompatible with mesenchymal stem cells (MSCs). (**A**) Quantitative analysis of DNA concentration of MSCs cultured in monolayer with IL-6 and IL-6 + BMP-2. Data are means ± SD; *n* = 8. (**B**) Quantification of osteogenic differentiation of MSCs cultured in monolayer with IL-6 and IL-6 + BMP-2 by measuring alkaline phosphatase (ALP) expression at day 21. ns = no significance. Data are means ± SD; *n* = 8. (**C**) Compressive moduli of GelMA at varying Irgacure concentrations and UV exposure times: low 0.15% IG—1 min (~3 kPa), medium 0.15% IG—2 min (~15 kPa), and high 0.30% IG—5 min (~30 kPa). Data are means ± SD; *n* = 3–4. (**D**) Representative live/dead images of hMSC viability with calcein-AM (green) to indicate intracellular esterase activity and propidium iodide (red) to indicate the loss of plasma membrane integrity. Scale bar represents 100 μm.

Figure 4 characterizes the DNA content and ALP activity from monolayer cultures treated with osteogenic media, IL-6, or IL-6 + BMP-2 (Figure 4A,B), alongside GelMA hydrogel compressive moduli and MSC biocompatibility (Figure 4C,D). The quantitative analysis of the DNA content and osteogenic differentiation of MSCs was consistent across all monolayer treatment groups (Figure 4A,B). The compressive modulus of GelMA hydrogels was tuned by varying Irgacure (IG) concentration and UV exposure time (Figure 4C). Specifically, low-, medium-, and high-stiffness gels exhibited compressive moduli of 3.3 ± 1.1 kPa, 14.7 ± 1.0 kPa, and 32.1 ± 2.4 kPa, respectively. Qualitative assessment of cell viability was satisfactory for both medium (0.15% IG—2 min exposure) and high (0.30% IG—5 min exposure) stiffness gels (Figure 4D). These findings underscore the biocompatible properties of GelMA and the ALP and DNA activity of monolayer controls.

**Three-dimensional culture attenuates pro-inflammatory and enhances anti-inflammatory hMSC secretome in response to combined IL-6 and BMP-2 stimulation.**

**Figure 5:**
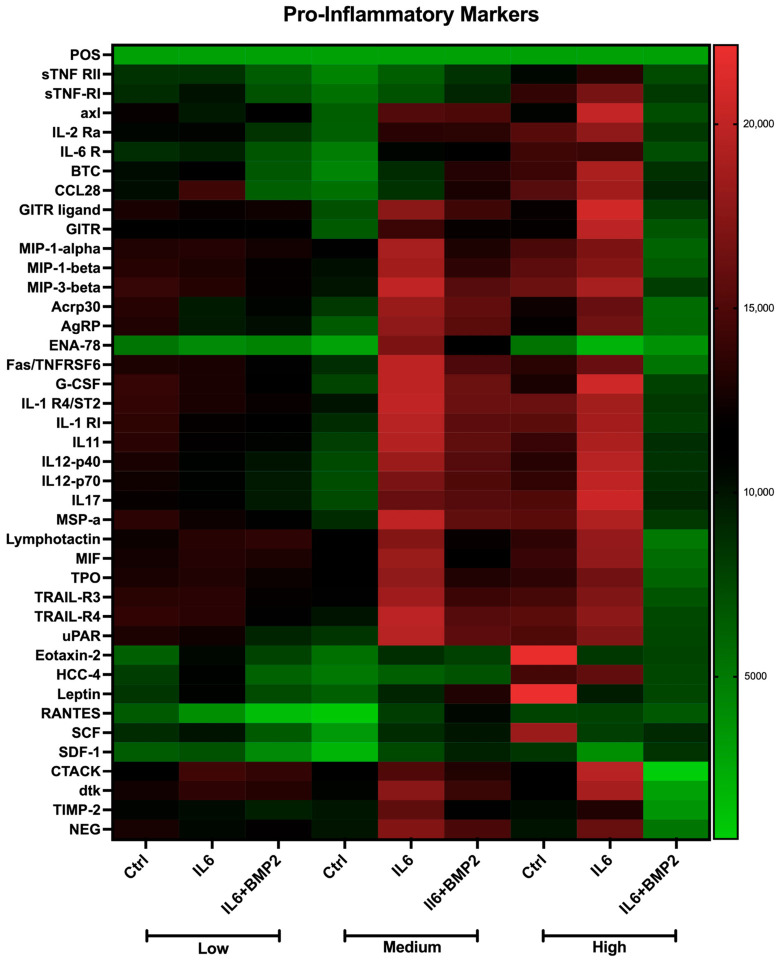
Three-dimensional culture results in attenuation of human mesenchymal stem cell (hMSC) pro-inflammatory markers in vitro when IL-6 and BMP-2 are combined. A heat map of the secretome protein expression of inflammatory cytokines from hMSCs in low-, medium-, and high-stiffness hydrogels. *N* = 6; samples were pooled together for each group. Green color depicts low levels, and red depicts high levels of expression. POS = positive control spots, which were used as the internal control/baseline.

The incorporation of MSCs into GelMA (Figure 5) was associated with reduced production of inflammatory cytokines, detected with IL-6 and BMP-2 co-treatment, observed using TCP (Figure 2). Figure 5 highlights the impact of GelMA on the inflammatory secretome profile of MSCs treated with osteogenic media and addition of either IL-6 or IL-6 + BMP-2. The heat map demonstrates distinct changes in the MSC secretome profile after encapsulation in gels with varying stiffnesses. All three stiffness levels, especially the high-stiffness gel, resulted in suppression of pro-inflammatory markers in the IL-6 + BMP-2 groups compared with IL-6 alone. Key pro-inflammatory chemokines, including MIP-1α, MIP-1β, RANTES (CCL5), and ENA-78 (CXCL5), were attenuated under GelMA conditions, indicating reduced leukocyte recruitment and inflammatory signaling. These results may imply that GelMA can suppress the hyperinflammatory secretome observed when culturing MSCs with both IL-6 and BMP-2.

**Figure 6:**
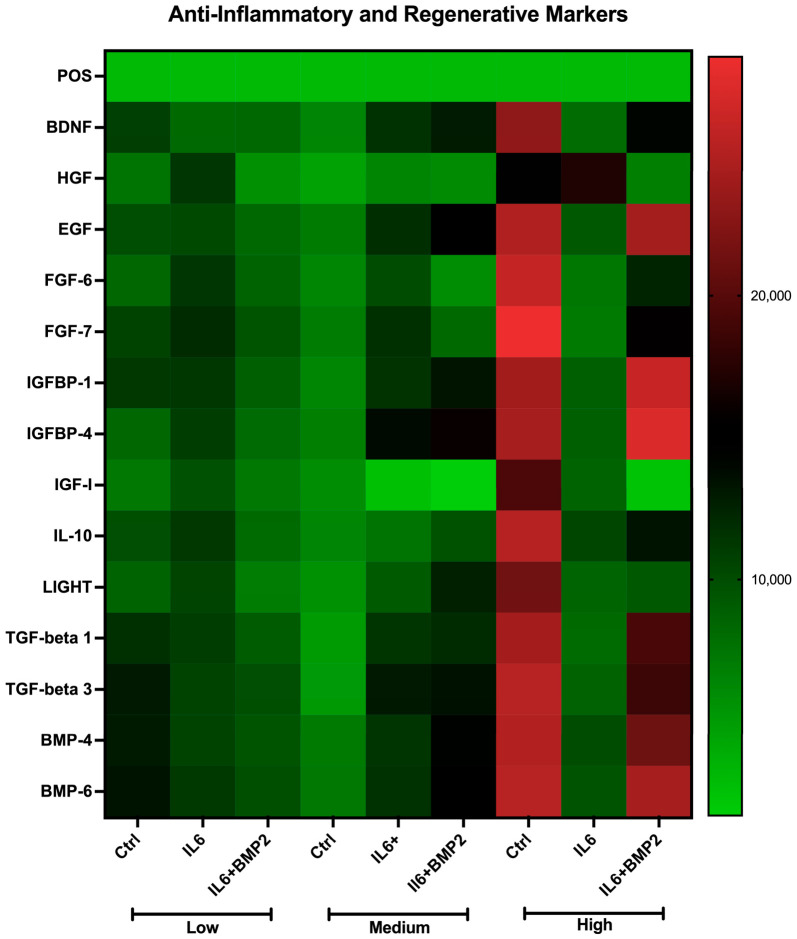
High-stiffness gelatin methacryloyl (GelMA) results in increased expression of anti-inflammatory markers on human mesenchymal stem cells (hMSCs) in vitro in the IL-6 + BMP-2 group. A heat map of the secretome protein expression of inflammatory cytokines from hMSCs in low-, medium-, and high-stiffness hydrogels. *N* = 6; samples were pooled together for each group. Green color depicts low levels, and red depicts high levels of expression. POS = positive control spots, which were used as the internal control/baseline.

Figure 6 depicts the heat map representing the anti-inflammatory and regenerative secretome profile of MSCs incorporated into different gel stiffnesses after exposure to IL-6 or IL-6 + BMP-2. While GelMA suppressed pro-inflammatory markers (Figure 5), anti-inflammatory and regenerative markers were upregulated. After MSC treatment with IL-6 and BMP-2, key cytokines, IL-10 (*p* = 0.001), TGF-β1, and TGF-β2, were upregulated in the presence of high-stiffness GelMA compared with IL-6 alone. Similarly, increased regenerative markers such as fibroblast growth factor (FGF-6) and FGF-7 were detected with culture in medium- and high-stiffness gels. Notably, the magnitude of upregulation increased with gel stiffness, suggesting that matrix stiffness may modulate MSC secretome responses under inflammatory conditions. Collectively, these findings suggest that the combination of 3D culture and entrapment in stiffer gels (~30 kPa) may attenuate inflammatory signaling while promoting anti-inflammatory and regenerative factor expression in MSCs.

**Medium-stiffness g** **elatin methacryloyl (GelMA) increased alkaline phosphatase (ALP) activity in IL-6 + BMP-2-treated MSCs.**

**Figure 7:**
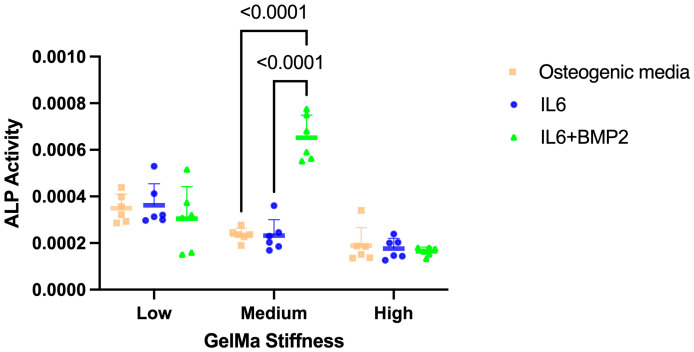
MSCs entrapped in medium-stiffness gelatin methacryloyl (GelMA) exhibit increased alkaline phosphatase (ALP) activity when treated with IL-6 + BMP-2. Quantification of osteogenic differentiation of MSCs cultured with IL-6 and IL-6 + BMP-2 for all three GelMA stiffnesses using alkaline phosphatase at day 21. Data are means ± SD; *n* = 6.

ALP activity of MSCs at each GelMA stiffness (low, medium, and high) was measured in the osteogenic media (control), IL-6, and IL-6 + BMP 2 groups. ALP levels were highest in the medium-stiffness gel after IL-6 + BMP-2 exposure (Figure 7). These results show that GelMA stiffness can affect the osteogenic differentiation of MSCs in vitro.

## 4. Discussion

Fracture healing is a tightly regulated biological process that depends on a coordinated inflammatory response robust enough to initiate repair yet sufficiently restrained to permit progression to regeneration and remodeling. Dysregulation of the inflammatory process, particularly in the setting of severe trauma, compromises fracture healing [4]. Elevated IL-6 and systemic inflammation have been linked to delayed repair outcomes in animal models of combined injury [12]. Additionally, clinical use of BMP-2 is associated with a range of adverse events, including postoperative inflammation and increased risk of heterotopic ossification after fracture [24,25]. In this study, we observed how IL-6 and BMP-2 modulate the inflammatory and osteogenic secretome of human mesenchymal stem cells, and whether encapsulation in gelatin methacryloyl (GelMA) hydrogels could attenuate inflammation while preserving osteogenic potential. Our findings demonstrate that while IL-6 increases inflammation and negatively impacts osteogenesis, IL-6 and BMP-2 together induce an exacerbated dysregulated immune secretome characterized by simultaneous pro- and anti-inflammatory signaling. Incorporation of hMSCs into GelMA, particularly at higher stiffnesses, attenuates this hyperinflammatory response while supporting osteogenic differentiation.

IL-6 is a pleiotropic cytokine with well-established roles in both immune activation and bone biology. In the present study, an IL-6 concentration of 1 ng enhanced alkaline phosphatase activity in hMSCs. However, prolonged exposure resulted in suppression of ALP, suggesting that sustained IL-6 signaling impairs osteogenic differentiation. Persistent elevation in IL-6, as observed in polytrauma or chronic inflammatory states, may, therefore, play a large role in disruption of the normal transition from inflammation to fracture repair. Our previous study highlights this disruption, particularly hyperinflammation and lack of transition to the repair phase, in a murine polytrauma model assessing fracture healing in the setting of concomitant chest trauma [4].

The interaction between IL-6 and BMP-2 further amplified these effects. While BMP-2 is a potent osteoinductive growth factor for bone regeneration, its clinical use has been limited by inflammatory complications [25]. Our data demonstrate that co-treatment with IL-6 and BMP-2 induces a pronounced shift in the hMSC secretome toward a state of immune dysregulation, characterized by upregulation of pro-inflammatory factors such as MIP-1α, MIP-1β, MIP-3β, IL-1, TNF-α, and GM-CSF, alongside increased expression of anti-inflammatory and regenerative mediators, including IL-10, TGF-β, and VEGF. Prior studies have similarly reported that BMP-2 treatment is associated with increased secretion of both pro-inflammatory cytokines (e.g., IL-1β and TNF-α) and anti-inflammatory mediators (e.g., IL-10), indicating that BMP-2 induces a complex and dysregulated immune response rather than a purely pro-inflammatory state [13].

Given the mechanosensitive nature of MSCs, we next investigated whether matrix stiffness could attenuate this immune dysregulation. GelMA hydrogels supported hMSC viability and early osteogenic differentiation across all gel stiffnesses tested, confirming their biocompatibility. Notably, incorporation of hMSCs into hydrogels markedly decreased the pro-inflammatory secretome induced by IL-6 and BMP-2 co-treatment. This effect was most pronounced in the higher stiffness group, which suppressed key chemokines involved in leukocyte recruitment, including MIP family members, RANTES, and ENA-78. These findings suggest that matrix stiffness can shape the inflammatory behavior of MSCs. Some studies have also indicated that GelMA can reduce the MSC inflammatory cytokine expression profile [26]. Donaldson et al. [27] found that TNF-α expression was significantly decreased in GelMA culture after lipopolysaccharide treatment was given to induce an inflammatory response [27]. Other studies suggest that addition of other drugs or bioactive factors to GelMA is needed to achieve substantial immunoregulatory function [28,29]. Based on the results of this study, the hydrogel may play a large role in modulating the host immune response by reducing pro-inflammatory signaling after BMP-2 and IL-6 exposure. Future studies should move beyond descriptive secretome profiling to mechanistically interrogate the intracellular signaling pathways that mediate matrix stiffness-dependent immunomodulation. Specifically, analysis of potential mechanoregulation pathways, including IL-6/JAK/STAT3 axis and NF-κb, which are known to be key modulators of the immune response, should be investigated [30,31]. The YAP/TAZ mechanotransduction pathway is well known to regulate MSC fate and may also be studied in the context of BMP-2 and IL-6 [32]. It is also important to determine if similar behavior is seen in hydrogels formed from other materials. Given that IL-6 peaks at 6 h, delaying BMP-2 administration until after the peak may help mitigate the hyperinflammatory and dysregulated secretome. Alternative approaches for locally reducing IL-6 levels during BMP-2 administration may also help alleviate the exacerbated inflammatory signaling with co-exposure.

Osteogenic, pro-, and anti-inflammatory factor outcomes exhibited a distinct dependence on matrix stiffness. Medium-stiffness GelMA resulted in the highest ALP activity in MSCs exposed to IL-6 and BMP-2, while high-stiffness GelMA resulted in the most robust anti-inflammatory immune secretion profile. Notably, the finding that medium stiffness (~15 kPa) optimized ALP activity, an early indicator of osteogenic differentiation, while higher stiffness (~30 kPa) enhanced anti-inflammatory signaling supports the concept that osteogenesis and immunomodulation may have different optimal stiffnesses. These findings align with studies suggesting matrices with stiffnesses from 11 to 30 kPa promote osteogenesis [20], yet others report that a higher degree of crosslinking and stiffness is correlated with greater osteogenic differentiation of MSCs [33,34,35]. In contrast with these findings, Zhuang et. al. [36] observed that low-stiffness GelMA (3.4 kPa) enhances MSC immunosuppressive signaling and supports the development of macrophages with anti-inflammatory features in vitro relative to stiff matrices [36]. These conflicting findings suggest that GelMA immunomodulation and osteogenic properties may be context-dependent, and introduction of IL-6 and BMP-2 may elicit a specific MSC response in combination with GelMA. Future studies with comprehensive datasets explicitly profiling a full panel of pro- and anti-inflammatory cytokines from MSCs are needed to help confirm these data. Additionally, an important limitation of this study is the inherent coupling between hydrogel stiffness and mesh size, as increased crosslinking in stiffer gels reduces pore size. Consequently, differences in matrix stiffness may also influence the diffusion of soluble factors and secreted cytokines, potentially confounding the observed effects on MSC behavior. Furthermore, only the initial compressive moduli of GelMA hydrogels were reported in this study. Hydrogel degradation as a function of crosslinking degree and in the presence of an inflammatory environment should be explored in future work.

These findings have important implications for the design of biomaterials used in fracture healing, especially in polytrauma settings. Mechanically tuned GelMA scaffolds may serve as regulators of the local immune environment, mitigating the inflammatory side effects of BMP-2, offering a strategy to reduce complications while preserving bone regeneration. However, this study has some limitations. Cytokine profiling was performed on pooled conditioned media samples (*n* = 6 per group) without independent biological replicates for array analysis, which limits the robustness of our conclusions regarding the inflammatory profiles. Future studies should incorporate additional biological replicates at multiple time points (24 h, 3, 7, 14, and 21 days) to measure cytokines and provide a more comprehensive understanding of the temporal evolution of the MSC secretome in response to IL-6 and BMP-2 stimulation. Furthermore, compressive modulus measurements were performed on a limited number of samples (*n* ≥ 3 per group), which reduces statistical power and may increase variability. Larger replicate numbers would strengthen the reliability and generalizability of the reported mechanical properties. Additionally, as an in vitro model, this study does not capture the full complexity of the fracture microenvironment, including interactions between MSCs and immune cells such as macrophages and neutrophils. Future studies incorporating co-culture with macrophages to assess polarization, in vivo fracture models, and mechanistic interrogation of mechanotransduction pathways will be necessary to further elucidate how matrix stiffness modulates inflammation and regeneration after BMP-2 treatment. It is recommended that studies focus on high-stiffness GelMA, given that 30 kPa best attenuated the hyperinflammatory effects of BMP-2 and IL-6 while preserving osteogenesis.

## 5. Conclusions

The findings of this study demonstrate that IL-6 and BMP-2 together induced a dysregulated immune secretome in hMSCs, characterized by increased pro- and anti-inflammatory signaling that may impair effective fracture healing. Incorporation of MSCs into higher-stiffness GelMA hydrogels attenuated this hyperinflammatory response while preserving osteogenesis. Together, these results highlight the potential of mechanically tuned GelMA to modulate immune responses. Future co-culture models and in vivo studies are needed to assess whether high-stiffness GelMa can improve bone regeneration in challenging trauma scenarios.

## Data Availability

The original contributions presented in this study are included in the article. Further inquiries can be directed to the corresponding author.
